# Redressing the gender imbalance: a qualitative analysis of recruitment and retention in Mozambique’s community health workforce

**DOI:** 10.1186/s12960-020-00476-w

**Published:** 2020-05-24

**Authors:** Rosalind Steege, Miriam Taegtmeyer, Sozinho Ndima, Celso Give, Mohsin Sidat, Clara Ferrão, Sally Theobald

**Affiliations:** 1 Liverpool School of Tropical Medicine, Pembroke Place, Liverpool, L3 5QA United Kingdom; 2 Universidade Eduardo Mondlane, 3453 Avenida Julius Nyerere, Maputo, Mozambique

**Keywords:** Mozambique, Community health workers, Gender, Agentes Polivalentes Elementares, Moçambique, Agentes Polivalentes Elementares, Trabalhadores comunitários da saúde, Gênero

## Abstract

**Background:**

Mozambique’s community health programme has a disproportionate number of male community health workers (known as Agentes Polivalentes Elementares (APEs)). The Government of Mozambique is aiming to increase the proportion of females to constitute 60% to improve maternal and child health outcomes. To understand the imbalance, this study explored the current recruitment processes for APEs and how these are shaped by gender norms, roles and relations, as well as how they influence the experience and retention of APEs in Maputo Province, Mozambique.

**Methods:**

We employed qualitative methods with APEs, APE supervisors, community leaders and a government official in two districts within Maputo Province. Interviews were recorded, transcribed and translated. A coding framework was developed in accordance with thematic analysis to synthesise the findings.

**Findings:**

In-depth interviews (*n* = 30), key informant interviews (*n* = 1) and focus group discussions (*n* = 3) captured experiences and perceptions of employment processes. Intra-household decision-making structures mean women may experience additional barriers to join the APE programme, often requiring their husband’s consent. Training programmes outside of the community were viewed positively as an opportunity to build a cohort. However, women reported difficulty leaving family responsibilities behind, and men reported challenges in providing for their families during training as other income-generating opportunities were not available to them. These dynamics were particularly acute in the case of single mothers, serving both a provider and primary carer role. Differences in attrition by gender were reported: women are likely to leave the programme when they marry, whereas men tend to leave when offered another job with a higher salary. Age and geographic location were also important intersecting factors: younger male and female APEs seek employment opportunities in neighbouring South Africa, whereas older APEs are more content to remain.

**Conclusion:**

Gender norms, roles and power dynamics intersect with other axes of inequity such as marital status, age and geographic location to impact recruitment and retention of APEs in Maputo Province, Mozambique. Responsive policies to support gender equity within APE recruitment processes are required to support and retain a gender-equitable APE cadre.

## Resumo

O programa de saúde comunitária de Moçambique tem um número desproporcional de agentes comunitários de saúde – Agentes Polivalentes Elementares (APEs) – do sexo masculino. O governo pretende aumentar a proporção de mulheres para 60%, para melhorar indicadores de saúde materna e infantil. Este estudo explora os processos de recrutamento dos APEs e como estes são moldados por normas de gênero; e como os papéis e relações de gênero influenciam a experiência e retenção dos APEs na província de Maputo, Sul de Moçambique.

Recorreu-se a métodos qualitativos para selecionar de forma propositada APEs, supervisores dos APE e líderes comunitários. As entrevistas foram gravadas, transcritas e traduzidas. Uma grelha de codificação foi desenvolvida de acordo com a análise temática para sintetizar os resultados.

Realizamos entrevistas em profundidade (*n* = 30), informante chave (*n* = 1) e discussões em grupos focais (*n* = 3) para captar as experiências e percepções dos processos de recrutamento. As estruturas decisórias intrafamiliares, implicam que as mulheres podem experienciar barreiras na adesão ao programa dos APE, exigindo frequentemente anuência dos esposos. Os treinamentos fora das comunidades representam aprendizagem em ambiente diferente, todavia, as mulheres relataram dificuldades em deixar as suas responsabilidades familiares, e os homens desafios em prover assistência as suas famílias. Estas dinâmicas agudizaram-se para mães solteiras com o papel de provedoras e de principais cuidadoras. A atracão do programa por gênero revelou que as mulheres tendem a abandonar o programa quando se casam, e os homens a abandonarem quando são oferecidos melhores empregos. A idade e a localização geográfica demonstram que os APEs mais jovens buscam oportunidades de emprego na vizinha África do Sul, e os mais velhos tendem permanecer no programa.

Sugerimos que as normas de gênero e a dinâmica de poder se cruzam com outros eixos de desigualdade, como estado civil, idade e localização geográfica, para influenciarem o recrutamento e a retenção dos trabalhadores comunitários de saúde. O fortalecimento dos sistemas de saúde requer políticas mais equitativas e sensíveis para apoiarem a equidade de gênero nos processos de recrutamento e manter dos APEs.

## Introduction

### Community health workers’ gendered experiences

Community health workers (CHWs) are increasingly relied upon to supplement human resources for health shortages in low- and middle-income countries. CHWs occupy a unique interface position between communities and the health system and can reach marginalised communities. Unlike other cadres, they do not operate out of an institutional space, but instead operate within the gendered boundaries of their communities [1]. Though gender norms and power relations are highly contextual, growing evidence demonstrates similarities in how gender dynamics influence CHWs at the individual, community and health system levels. At the individual level, male family support can be a critical factor in whether women are able to take on the role in contexts such as in Afghanistan [2], Bangladesh [3], Pakistan [4] and Kenya [5]. At the community level, cultural restrictions around women’s movement in some contexts obstruct both women’s access to healthcare and CHWs’ access to client’s homes [2, 6–8]. At the health system level, female CHWs have limited opportunities for career progression and to input into policy-making [8–12]. Further, whether a programme is remunerated or not brings nuanced gendered challenges. Voluntary programmes reinforce the gendered segregation of the caring role, where women and girls constitute most of the informal care workforce [13, 14] and may be difficult for men, often perceived as breadwinners, to commit to [1]. Yet, these supply-side gender dimensions are not reflected in policies which establish and guide CHW programming. This limits the provision of high-quality services and may mean CHW programmes reinforce, rather than challenge, gender inequity [1].

**Table:** 

***Box 1: Key definitions***	
**Gender**—Gender is defined as the ‘socially constructed roles, behaviours, activities and attributes that a given society considers appropriate for men and women and people of other genders'.	
**Gender analysis**—A critical examination of how differences in gender roles, activities, needs, opportunities and rights/entitlements affect women, men, girls and boys in certain situations or contexts.	
**Gender norms—**Accepted attributes and characteristics of being a woman or a man (ideas of how men and women should be and act) at a particular point in time for a specific society or community. They are internalised early in life and are used as standards and expectations to which women and men should conform and result in gender stereotypes.	
**Gender roles—**Refers to what males and females are expected to do (in the household, community and workplace) in a given society.	
**Gender relations—**Refers to social relations between and amongst women and men that are based on gender norms and roles. Gender relations often create hierarchies between and amongst groups of men and women that can lead to unequal power relations, disadvantaging one group over another.	
**Gender transformative—**Addresses the causes of gender-based health inequities by including ways to transform harmful gender norms, roles and relations. The objective is to promote gender equality and foster progressive changes in power relationships between women and men.	
Adapted from the United Nations. Gender Statistics manual. Available at https://unstats.un.org/unsd/genderstatmanual/Glossary.ashx	

### Mozambican context

Mozambique’s limited health workforce is one of its greatest barriers to the provision of quality services [15, 16]. Recognising this, the Mozambican government aims to expand their CHW programme from the current 16% coverage to 35% by 2024 [17]. CHWs in Mozambique are known as Agentes Polivalentes Elementares (APEs), meaning ‘essential [or elementary] multi-purpose agents’, and this is the term we will use in this paper. Following independence in 1975, Mozambique has promoted a health policy based on the principles of broad and equitable access to health services through sustained expansion of the primary healthcare system [18, 19]. In 1978, the APE programme was introduced to meet the needs of rural communities experiencing limited access to healthcare services. Following 16 years of civil war (from 1976 to 1992), the APE programme was left without adequate supervision or technical support and was suspended in 1989 [16, 17, 20]. In 2010, Mozambique launched a revitalised community health programme.

The APE programme recommends that the time is divided between curative services and health promotion activities 20:80, respectively. APEs undergo a four-month training programme that reflects this, with emphasis on health care maternal, neonatal and child health, as well as first aid, recognition of common diseases—malaria and diarrhoea—and referral to health units [21]. Curative services are limited to testing and treatment for malaria, treatment of diarrhoea, provision of antibiotics for acute respiratory infections in children and first aid. The policy states that the APEs should come from the community that they serve. One APE should be recruited from each community of 500 to 2000 inhabitants [21]. APEs are not formally employed, but sign an agreement as ‘volunteers’, which qualifies them to a monthly subsidy of 1200 meticais (approximately US$ 20 per month, the poverty line is US$ 1.90 daily), which can be withheld for incomplete or delayed reports, and access to free healthcare at the local health centre [19]. APEs do not qualify for direct payment as employees by the government, because the public service law stipulates a minimum academic qualification of grade 10 [20]. This excludes APEs as the APE programme only requires minimal literacy and basic arithmetic competencies [20].

Mozambique sees a disparity in labour force participation between males (75.8%) and females (26.3%) [22], which is reflected in the sex breakdown of APEs. As of December 2018, there were 1453 females to 3334 males serving as APEs [17]. This is despite targets set by the National APEs Coordination Office for a greater number of females in the role. In the 2011 Operational guidelines for the APE programme, under selection, the following guidance is stated ‘The selection of women should be encouraged. 60% of the candidates must be women, due to their importance in education, and health care in the community.’ [23].

Mozambique currently ranks 120 on the gender development index [24]. Despite a strong political commitment to gender equality, health indicators for women and girls in Mozambique show pervasive discrimination [25]. The maternal mortality ratio in the country stands at 490 per 100 000 live births [24]. Women have also been shown to have limited autonomy over their maternal health decisions and may be prevented from leaving the house to access care [25–27]. Across contexts, using female CHWs is one strategy to work within gender roles around maternal and child caregiving and circumvent women’s limited mobility [10, 28, 29]. Within the Mozambican context, it is also perceived that women have a greater cultural ability to deal with maternal and child health issues [17] and that the preponderance of male APEs may deter women from seeking care for newborns, as men are excluded from care after birth [20]. Thus, the unbalanced ratio of male to female APEs requires attention in terms of their ability to access key target groups and from a human resources equity perspective.

Despite the stated intention to increase the numbers of female APEs, there are few available studies that explore the reasons behind this persistent gender imbalance, and none with an explicit gender analysis. One rationale, as cited in the unpublished 2018 strategy document, is the lower level of schooling amongst the female population [17] (primary school enrolment in 2017 was 76% for females and 82% for males—attendance varies by intersecting inequalities such as location and wealth [30]). A recent study with national-level key informants also highlighted men’s relatively higher literacy rates (71% for adult males vs. 43% for females [30]) and community selection processes favouring young men because they feel that men are more deserving of paid work and the opportunity for advancement [20]. These highlight a need for further exploration of gender dynamics at play for Mozambique’s APEs.

### Study aim

**Fig. 1:**
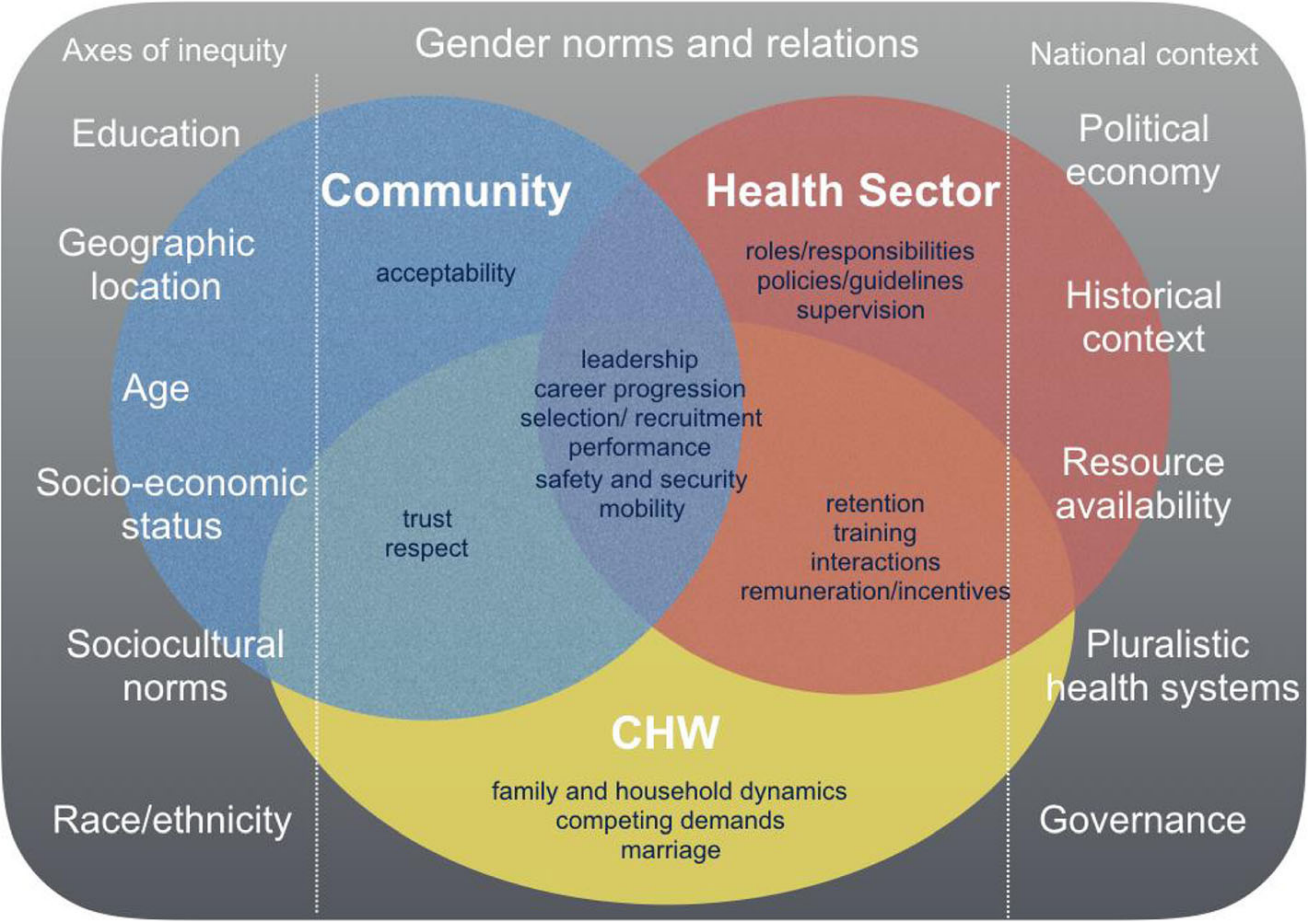
Conceptual framework, adapted from [1]

This study was nested within the REACHOUT consortium [31]. The aim of this study was to explore how gender norms, roles and relations influence the APE experience, and identify priority areas to support the recruitment of female APES and improve retention of APEs. A conceptual framework developed by Steege et al. [1] (Fig. 1) maps out the areas where gender norms and power relations can impact on CHW programmes across the individual, community and health system levels and was used to inform the design and analysis of this study. It highlights the importance of community dynamics, which play a role in the selection of candidates and family dynamics that shape decisions to join and remain in the role. It also illustrates how axes of inequity (to the left of the conceptual framework) and contextual factors (to the right) influence how gender is experienced more broadly. Intersectional analyses, which take into account the unique experiences that arise at the interaction between gender and different power asymmetries such as ethnicity, religion, socio-economic status and age [32, 33], are currently lacking from the Mozambican CHW literature. This framing is crucial in understanding the way gender plays out for APEs who are often at the lower end of the gender-class-socio-economic hierarchy. Although this study is not an explicit intersectional analysis, we have aimed to portray the unique experiences that arise at the intersections of different power asymmetries.

## Methods

### Recruitment and data collection

Qualitative methods were selected to understand the reasons behind the gender imbalance and explore social and gender norms within communities. Qualitative methods allow exploration of the processes of recruitment and retention of CHWs, which may involve complex social and political processes linking CHWs’ experiences, values and desires, and relationships with the institutions that recruit them [34]. Methods included face-to-face in-depth interviews and focus group discussions with a mix of APEs, APE supervisors, community leaders and a key informant from the Ministry of Health. Participants were purposively selected to ensure representation based on geographical location, sex and job experience.

**Fig. 2:**
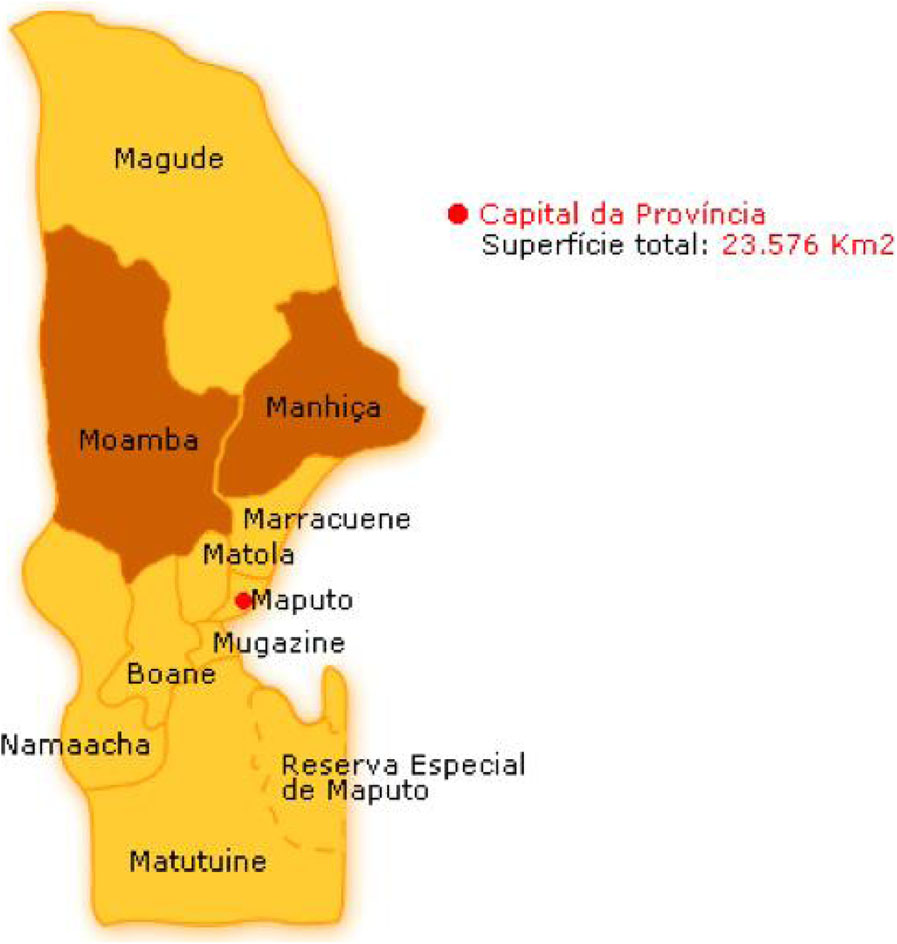
Map showing the study districts of Moamba and Manhica, north of Maputo

Interviews were conducted in two convenience sampled districts within Maputo Province that were part of the REACHOUT consortium study sites: Moamba and Manhiça (Fig. 2). In contrast to the rest of the country, there are actually more females in the role than men in these study sites (Moamba—nine males, 16 females; Manhiça—10 males, 30 female). Unfortunately, it was not logistically possible to conduct this study in districts that are more representative of the wider gender dynamic in Mozambique. As this is a qualitative study, we will be careful not to generalise the findings to the rest of the country. Further, it is still relevant to explore why there are more females in this part of the country and what their, and indeed their male counterparts, gendered experiences are within the role.

The districts are mainly rural with established revitalised APE programmes and similar epidemiological profiles; however, their health network remains insufficient to meet the needs of the population [35]. A key similarity is their proximity to South Africa which brings additional livelihood opportunities from other districts in the country. A key difference between the districts is that Moamba is inland (bordering South Africa), whereas Manhiça is a coastal town. In Manhiça, residents are employed by the sugar and rice industry, as well as engaged in fishing and informal trade and migrant labour in South Africa. In Moamba, residents are engaged in agriculture and informal trade and migrant labour in South Africa.

A female research assistant (CF), experienced in qualitative techniques, was recruited to ensure a gender-balanced research team in case of sensitive questions. She was trained in qualitative interviewing techniques (e.g. open-ended questions and probes), the research objectives and using the topic guides. Most interviews were conducted in Portuguese by CF. Interviews with male community leaders were conducted in the local language (Xi-Changana) by experienced qualitative researchers, SZ and CG, who were also trained.

Interview topic guides, informed by the conceptual framework (Fig. 1), explored the gendered norms, roles and relations within the community that impact on health system processes of recruitment, retention and training of APEs, and the gendered experiences of APEs across the individual, community and health system levels. The interview topic guides (see Appendices 1, 2, 3, 4 and 5) were piloted in the field and refined. Interviews were conducted in communities and district health centres. These were scheduled in private spaces to avoid any distraction and to ensure the confidentiality of respondents. They were scheduled at a time convenient to the respondents and were recorded using digital Dictaphone devices.

### Analysis

The recordings were transcribed verbatim and translated into English. Back translation from English into Portuguese was conducted in a small sample of transcripts to assure the accuracy of the translation.

Transcripts were read and reread to identify emergent themes. A coding framework was developed in accordance with a thematic analysis [36, 37]. An inductive approach was primarily used, but some a priori themes were informed by the Steege et al. conceptual framework and included domains around training, recruitment and selection processes. Transcripts were uploaded to the software NVivo 11, where they were coded. To improve trustworthiness, data from individual interviews and focus group discussions were compared in order to triangulate the findings [38].

### Ethical considerations

Written informed consent was obtained from all participants. Ethical approval was obtained from the Liverpool School of Tropical Medicine no. 16-022 and the Institute of Bioethics in Health, Faculty of Medicine/Central Hospital of Maputo no. CIBS FM&HCM/45/2014.

## Results

In-depth interviews (*n* = 30), key informant interviews (*n* = 1) and focus group discussions (*n* = 3) captured gendered experiences of APEs, community leaders and health system staff (Table 1). These are presented here via the themes from the conceptual framework.

**Table 1:** Qualitative interviews by participant type and district

Participants	IDI		FGD		KII
Ministry of Health official					1× male
District level	Moamba	Manhiça	Moamba	Moamba	
APE	3× males 4× females	3× males 4× females	1× mixed sex^a^ (6 participants)	1× male (8 participants) 1× female (8 participants)	
APE supervisor	1× male 2× females	1× male 2× females			
District supervisor^b^	1× male	1× male			
Community leaders	3× males 1× female	3× males 1× female			
Total	*N* = 30		*N* = 3 (22 total participants)		*N* = 1

^a^Male and Female APE FGD merged due to limited numbers

^b^In text quotes are labelled as ‘supervisor’ to adhere to confidentiality

### Experiences of selection and recruitment

#### Recruitment motivations

APE respondents reported an intrinsic motivation for joining the APE programme, to help their community due to the limited services available or as a way to serve the country. This was common across both districts and genders. Other participants cited extrinsic reasons for joining the APE programme: because they were selected by the community, because they want to go on to become a nurse or because they were not accepted into formal health positions. Some spoke of the lack of employment opportunities and suggest people joined ‘to have an occupation so that they don’t remain without doing anything’ or to hold a position of responsibility—which brings status. However, supervisors articulated a financial motive for many people to join the programme, linked with pressures of modern-day life:

*I think in the early days, I think people wanted to be APEs to help even, to help others in those times, but in nowadays, in the present moment, I think they want to be APE, I think it's more to get something like money, to work*. [Supervisor IDI Manhiça, female]

#### Selection process

Literacy requirements may not only discriminate against female candidates, but also those whose education was affected by the civil war and could not reach the required educational level. Despite this, one male APE was serving despite not finishing his education. This highlights how the historical-political context also influences who may become an APE.

The selection process was described by all groups of respondents to be led by community leaders. It was also stressed that scores are judged alongside the personality of the candidate and who will be best suited to serve the community. With regard to the selection by gender, participants suggested that there are equal opportunities for both males and females to be selected, and district supervisors spoke of the emphasis of ‘gender balance’ in the recruitment and selection of APEs. As aforementioned, in the study sites, there are more females in the role than men. This is in part due to the prioritisation of women as denoted in the selection policy, as well as employment opportunities for men in South Africa, which meant women were left alone in communities and had more autonomy over their own livelihood choices and adopted the traditionally male ‘breadwinning’ role.

…*the [Southern] provinces of Gaza, Maputo and Inhambane have a greater number of women. This is because… there are many women with husbands working in South Africa and as a way to take care of the family, women have to find something to do. This is the difference that we have, looking at the Central zone and North the woman has to take care of the house, the woman has little time, she cannot do anything, she is dependent on the man, the man is the one who determines if she is going to work… [in the South] …they adhere because their husbands are not there, they are out of the country working in the mines*… [KII MoH, male]

Traditional gender roles that ascribe domestic duties and caring roles to females and outside labour to males also influenced community perceptions and selection of candidates.

*It’s just because women have more time. Looking at women’s side you will see that they are who have this ability to take care of us, and men have more activities to do and must not be more related to this work*. [Supervisor IDI Manhiça, male]

*I think women are the most helpful. There are a few helpful men, but the big problem is that some people take the job and, without giving any information, leave to South Africa, leaving the community to their fate. That's one of the reasons that made our choice fall on a woman*. [Community Leader IDI Manhiça, male]

It is stated in the operational guidelines that no form of favouring the candidates should be considered or accepted (e.g. religion, party, politician, family) [23]. Nevertheless, examples of nepotistic selection were also given by respondents with regard to the recruitment of men which was associated to their perceived need for income-generating opportunities due to the provider role.

*A: …I worked with the father of this young man, he was my servant [health position] and so when this request came I did not go far, I came to get him right here.* [Community Leader IDI Manhiça, male]

*…during the selection they have not noticed the [female] side of being more open, more welcoming. They have noticed the friendship side, family, it’s much more a chance I give my nephew… to be APE, since he does not work, being a man…* [Supervisor IDI Moamba, female]

#### Training programme

The four-month training that must be completed to become an APE is offered in a central location. APEs who live far away may only return home at weekends, if at all. This residential-style training creates difficulties for some candidates due to their gendered roles at home. Due to a ‘provider’ role, male APEs spoke of the challenges of providing food for their families at home. During the training, they only have access to the allowance provided by the health system as they are unable to engage in any additional income-generating activities that they could at home:

*If I have to leave here to be in another place, for four months, here at home what is going to be eaten? For example, because now where I am, I only have 10 kg [of rice], I am trying to at least increase again that 10 kg, or this woman [wife at home] is going to haunt you. How is it possible to work to increase that food with that little allowance they gave us?* [APE FGD Manhiça, male]

Experiences of training were also influenced by national context as shown to the right of the conceptual framework (Fig. 1). For one cohort of APEs, the difficulty of not being able to fulfil the provider role for their family during training was compounded by famine at the time of training. Although they faced difficulties during the training period, they spoke with defiance:

*In the family, when I leave, I’m the male there (laughter) so no one will be [disciplining] the children for four months. I do not know how I made it staying here because [home] was lacking food…when we were trained it was time of starvation…We also had problems of a lack of food here, if I had a [lunchbox] here sometimes I would carry it to a child at home, but here we had difficulties. But we faced them, we are here, we win. We are winners.* [APE, Male FGD Moamba]

Difficulties facing women who train away from home were linked to marital status. In the cases of single mothers, who were also serving a ‘provider’ role, the stress of not being at home to support and provide for children impacted their training:

*For me it was difficult because I am a mother, I am a father, I have a son who is seven years old… I even asked my sister to go stay with my son, sometimes the night at [8 pm] she sent me a [message] then I seek airtime to call, she just wanted to tell me that at home has no oil, no sugar, has nothing, I was not well trained because it was not always easy.* [APE Female FGD, Moamba]

These examples highlight the socio-economic vulnerability of APEs who risk the welfare of their families and endure stresses to complete the training. The reliance on family members was expressed by both male and female APEs, by women with regard to helping with childcare and by men with regard to helping with farming—emphasising the gendered divisions of labour common to the study sites.

Patriarchal norms that dictate women are not the primary decision-makers in the home meant married women had to seek their husband’s consent and support over their livelihood opportunities. Barriers existed for some women to obtain consent to train away from home, but also to work out of the home, giving up time dedicated to household duties for a perceived lack of financial contribution to the household.

*Q: Do their husbands agree to let their wives go to the training for four months?*

*R: That, indeed, happened. The APEs that were in the training with our APE, there were three ladies whose husbands did not like and so the [husbands] suspended them from the training. However, when we distributed the cattle offered by PATHFINDER to the other APEs, their husbands complained.*

*Q: What are the motives for men who have suspended their wives from APE training, other than what they have already mentioned related to lack of salary?*

*R: The main reason was related to home activities. They did not see the reason for leaving their homes for an activity that has no benefits.* [Community Leader IDI Manhiça, male]

In these instances, supervisors were recruited to speak to husbands to gain their support. Yet, it was perceived to be a common barrier; one male APE suggested health system staff should meet with husbands of married female candidates and only recruit those who had the support of their husbands from the outset, to avoid investing in training women who may later quit if ‘the husband starts to make problems’.

Despite the issues raised with residential training, participants still felt it was preferable in order to build a cohort. They also spoke of a sense of pride gained from going away and returning ready to serve your community. Men placed emphasis on the benefits of collective learning. For women, however, the benefits also came from being away from the distractions of home life, allowing for focus and punctuality, which speaks to their double burden of household and APE work.

### Experiences shaping retention of APEs

#### Remuneration

Gender and power relations impact on retention in complex ways. Although it was reported both men and women leave due to low subsidies, this appeared to be more pronounced amongst men and shaped by husbands in the cases of married women.

*Q: In your experience, why do people usually leave?*

*R: Money problems.*

*Q: Is it money for both men and women?*

*R: …It is money because other women sometimes have a husband and the husband lets her go to work and at the end of the month there is nothing, before we were eight months without receiving the subsidy and the husband becomes demoralised, it is preferable to leave because… they do not give you anything* [APE IDI Moamba, female]

It was reported that the male role as the ‘breadwinner’ meant that men would often leave to find better-paid work and were more disheartened about the subsidies being low or delayed.

*Q: You said men usually give up, what are the reasons men quit?*

*R: Because men are the head of the family and all responsibilities rely on them, such as providing money for children to go to school, food and many other things. So, the reasons for many to quit taking in account the amount we receive as subsidy while we have much work to do as APE.* [APE IDI Moamba, male]

The sentiment that the subsidy was not commensurate to the workload was expressed by many of the APEs both males and females. As well as a leading cause of attrition, limited remuneration was a source of difficulty and stress. In most cases, however, a sense of obligation to the community and limited livelihood opportunities within the rural settings kept many in the role.

*I have worked with many projects but never ceased to be an APE and I will not leave, so far, I want to continue, even without money… A person’s life is not bought.* [APE IDI Manhiça, female]

Female participants in Manhiça, in particular, spoke passionately about feeling exploited by the health system—describing how there is an influx of work from multiple non-governmental organisations (NGOs), but the monthly subsidy does not change to reflect this. The APEs feel they are working to support their country, but yet the country is not speaking up for their rights, describing themselves as ‘slaves of the ministry’.

*More and more NGOs coming with more work but no money for that. The first organisation that came in said will give 1200 [meticais to] APEs, and these new organisations that are coming in now are exploiting us, but we will not stop working… We are going to work because we are in our country, in the community, what we are doing is not only helping the community alone, we are helping the whole country… but the country is not giving us priority, it does not value our work… This NGO asks the State, we want APE, they should say ‘we have APE, these APEs are receiving the value of another NGO if you also want to work with APE you also have to give something!’ Does not mean anything working for five NGOs while I'm only receiving salary from one NGO.* [APE IDI Manhiça, female]

These quotes demonstrate the APEs had internalised the rhetoric of their work being ‘priceless’ and for the good of their community and country. Women spoke about feeling trapped—unable to earn money due to their low educational status, but at the mercy of the Ministry to offer training to improve it.

*And if the ministry cannot pay us because our level is so low it should train us more us more, increase our training, because we have the capacity to learn more… We are still available to receive more studies, to continue helping the population, but we have to see the subsidy because they are already exploiting us, we are slaves of the ministry, because even servants receive more than we do, but we are doing a lot of work…* [APE FGD Manhiça, female]

#### Career progression

Alongside minimal subsidies, minimal opportunities for new training and career progression are cited as a contributing factor to their sentiment, as illustrated above. Speaking of the opportunities for female empowerment and further education, female APEs felt it would be inspiring for communities to see an APE go on to a university—reflecting how the health system could not only help meet the educational goals for women but also aspirations for this cadre to progress into the formal labour market.

*We hope they will help us, maybe they may give us scholarship so we can increase our level. Don’t you one day want an APE like me, to graduate from university? Wouldn’t you guys like that? Hearing that an APE, a lady was an APE, now she has done a degree.* [APE FGD Manhiça, female]

Opportunities for training and education are also linked with policies for remuneration. The relatively low education level of this cadre means they do not qualify for formal paid jobs within the public sector. Currently, the level of education held by APEs fits the role being ‘voluntary’. However, this voluntary status also brings with it its own challenges in juggling APE work with paid opportunities. The voluntary status of the APE role allows for concurrent paid employment; however, in reality, the requirements of the APE work limit opportunities to earn additional income. APEs felt continuously on call and have to attend to patients and their supervisors on demand.

*…the APE accepted to help the community but did not accept to be exploited, now the APE is being exploited. They are violating the rights of the APEs… they made a doctrine, it says the APE has no right to receive much subsidy because we have the right to go to the farm, do other jobs… but when the supervisor calls to say ‘I am coming to your house, I come to do supervision’, that day even if you have another [job] you cannot leave the house, have to wait for the supervisor to come to do supervision then that day that you were not in that work that sustains you is missing… We are working and we are happy with our work, but our concern is only subsidy…* [APE IDI Manhiça, female]

Balancing time between APE work and additional income-generating work also had a gendered element and posed particular problems for male APEs. They felt if they took another job in the daytime, they would not have time for APE work as it was not deemed appropriate for them to attend to families at night and would be accused of ulterior motives.

*Subsidy demoralises us a lot, other APEs eventually get another job, so the boss who employs me, will not accept the rest of the time to work as an APE. For example, you can say that you enter here [at] 6:00, I will leave 18:00 hours, so already for the work of APE I will not do anything because I will leave at 18:00, I cannot do home visits at night, because they will say you are no longer working in health, you want our women (laughs) …We are not well with will, but we are working.* [APE FGD Moamba]

This demonstrates how policies surrounding remuneration can impact men and women differently on the ground. In this case, the need for additional income restricts men’s ability to carry out APE work effectively due to socio-cultural norms that limit their ability to conduct night visits—which may impact on their retention.

#### Family, marriage and household dynamics

It was reported that women often leave if they get married and follow their husbands to their home villages. Supervisors expressed that this can be a problem for the health system once an investment in training has been made, but the woman is no longer able to work. A young, female participant articulated a romanticised rationale for quitting the APE role to start a family and spoke of her aspirations to be able to start a family and provide for them. This highlights the importance of understanding APEs as individuals and not viewing them as a homogenised group. This is particularly relevant in the case of female CHWs where the dominant narrative may be of oppression. Instead, individual circumstances come together to shape individual choices.

*Q: Do you think that one of the reasons men tell women to quit is because they do not want them to have their own money, their financial independence?*

*R: No, that's not correct. For example, I am a girl, I am an APE, I am dating a boy from Gaza province, he wants to marry me, well, I can say I have reasons to accept him and quit APE work, but this decision is not about money, it’s because I love him, I want to have my own home and family, as well as conditions to get food and educate my child.* [APE FGD Moamba, female]

Still, it is not always the woman’s choice to quit APE work. Patriarchal norms that dictate that men are the primary decision-makers in the household were also reported to impact on married women’s ability to remain in the role. In this case, husbands’ disapproval was interlinked to APE programme policies—both in terms of the limited income that the role of APE brings in and in the lack of a dedicated health post—which was also cited by many community leaders and APEs as being something that would benefit their work. One male participant described a situation where women who use their home as a place of work could lead to disagreements at home, and even domestic violence.

*…Women give up because the husband has a decision at home, to this day there is this, there is a husband who has a decision at home. ‘You should stay here, instead of going to the farm, leave these people, or even leave work, leave these activities here, I say stay here at home.’ Others drink, smoke, these husbands are stubborn, for example, if I come home, I drank, I smoked and I find you, my wife, and there is someone having a test there, I can make you stop [treating them] there and prepare a meal for me at the table…as I am the one who decides here at home… she will quit (the APE) because she wants the save her marriage. He even announces this, ‘you know nothing, your income adds nothing here at home.’ She ends up giving up because no woman will like to always receive beating, punches, slaps and know that I am not gaining anything.* [APE FGD Moamba, male]

The ability to negotiate alternative outside livelihood opportunities is shaped by gender, age marital status and geographic position. Younger men and women were viewed as being less inclined to stay in the position and more likely to look for job opportunities outside of their communities. Though, it was articulated to be more pronounced amongst young men and associated with their gendered provider role.

*For example, this young man here [an example APE man] receives 1200 [meticais], will he build real home with 1200? His girlfriend wants artificial hair, but this 1200 is not enough, so, if this APE gets a good job he will definitely leave APEs work. If someone tells him that there’s a job opportunity in Inhambane province where he’ll get paid five or seven thousand he will definitely leave APEs work. On the other hand, for an old man it’s different because there are few opportunities due to the age, so old men are likely to remain in their APE work. It is difficult for any old man to quit. He can remain in his community.* [APE FGD Moamba, male]

*Well in my group there was a colleague after training worked a few months but ended up giving up, travelled to South Africa but she was not married, even was a young woman in her 20s, so I do not know what was her influence, eventually she abandoned and on her return she wanted to get involved again but she had already been replaced by another colleague.* [APE IDI Manhiça, male]

These examples highlight the need for an intersectional lens when thinking through gender issues, as multiple factors interplay with gender to create unique situations that mean one person may have the ability or desire for higher income that causes their attrition.

## Discussion

The role of CHW provides increased agency and status in communities and can be an empowering experience [8, 10, 28, 39–43]. Our findings also reveal the dynamic and conditional nature of this empowered position, which can be altered by policies and shaped by multiple intersecting factors. Both districts in our study area had greater numbers of women working as APEs, which is at odds to what is seen in the country as a whole and particularly the dynamic in the north of the country. This is an obvious limitation; however, exploring their experiences helps guide suggestions for the recruitment of women and encouraging retention of men and women. It further serves to highlight the importance of context: how policies play out within countries varies, not just between countries. Our findings showed differences not just by districts, but by individuals. APEs are not a homogeneous group, and gender, age, geographical location and marital status all converge to influence both recruitment and retention of APEs. The conceptual framework (Fig. 1) used to frame our results, highlights this complexity: APEs’ experiences were influenced by other axes of inequity, which were important in shaping APEs’ outside livelihood opportunities, and national context, which included the historical impact of war and resource availability during times of famine. We present recommendations for policy to support recruitment and retention based on our findings within key themes from the conceptual framework. These are summarised below in Table 2.

**Table 2:** Key areas for policy change based on factors influencing recruitment and retention from the conceptual framework relevant to our findings

Health system goals	Policy changes and rationale
**Recruitment factors**	
Training	Modular, flexible training options support both men and women who balance APE work with other paid employment opportunities and domestic obligations. This can also help to reduce issues around the length of training APEs face in being away from family obligations. Residential training can create a sense of solidarity but can be limiting for women with childcare responsibilities, and men who feel a duty to provide for their families. On-site childcare should be offered to support this.
Household dynamics	Community sensitisation programmes to encourage women to have autonomy in decision-making over livelihoods and challenge patriarchal norms.
Selection	Training with community leaders on gender and power relations as well APE roles, programme aims and importance of accountability to ensure women have equal opportunities for selection.
**Retention factors**	
Remuneration and social security	Remuneration for APEs commensurate with working hours and skills (in hand with strict selection criteria to ensure equal selection of women). This will help reduce attrition and improve motivation. Social security in the form of employment-based health insurance, a component of universal health coverage, should be provided. Contracts should ideally be issued to formalise labour rights for CHWs, such as maternity and paternity leave and holiday and sick pay. This provides security to APEs and does not discriminate against women for their reproductive roles.
Career progression	Sufficient educational opportunities to improve literacy for female CHWs who want to go on to further education would contribute positively to the development of communities. Sufficient sponsored courses for eligible female and male APEs to undertake further training to enter into the health system. Opportunities for a career structure, e.g. promotion to supervisor or senior APE. This should be considered alongside barriers women may face to get there and be accompanied by mentorship, supportive supervision and preferential selection of qualified female candidates until gender parity is achieved.
Working conditions	Provision of formal, stocked health posts for APEs to support the safety and legitimacy of the cadre. Sensitisation on gender-based violence with community members.

### Modular, flexible training

Experiences of training were heavily influenced by gender roles that dictate household chores and child-rearing be predominantly carried out by women. The four-month training programme away from home impacts some women who may find it difficult to eschew their domestic roles, which often include being the primary caregivers to children. Conversely, men’s ability to fulfil the ‘provider’ role for their family was challenged as they rely on the minimal subsidies set out by the Mozambican government and cannot take part in additional income-generating activities for these months. The expectation that the CHW role should fit in with income-generating activities not being met has also been reported in Ghana [44]. Experiences of the training programme were also shaped by intersecting factors—such as marital status. In the case of single mothers who take on both caring and ‘breadwinning’ roles for their families, the pressure to provide caused particular stresses during training. External factors such as political context and resource availability also influenced training experiences, as demonstrated by the discussion of famine at the time of training. The financial stresses taken on by APEs during training illustrates their economic and social vulnerability—something which has also been noted in work from Nicaragua and Ethiopia [40, 43]. This vulnerability further highlights their unique embedded position in communities, occupying the same socio-economic bracket as their neighbours, but with slightly elevated status.

To support the training of this cadre and ensure that men and women are not limited by their domestic roles, we suggest that residential training be accompanied by childcare provision and a formal salary, with an option to complete the training in shorter modules to allow for breaks at home. Modular learning would also support opportunities for further income generation, which limited men in particular. Residential, modular training with on-site childcare facilities has been rolled out in India to support the female CHWs (known as ASHAs) with childcare obligations, whilst building an immersive and empowering learning experience [10].

### Gender transformation within communities

Gendered intra-household bargaining and male decision-making power have been well documented with regard to limiting women’s autonomy over health-seeking behaviour in low- and middle-income countries, including Mozambique [26, 45–47]. Our research extends this literature to show how gendered intra-household bargaining also shapes APEs’ own experiences and their livelihood negotiations. In these cases, patriarchal hierarchies within the family impact both women’s access to healthcare and women’s choices in delivering healthcare. Married women, in particular, may have less power over their decision to become an APE as their often-limited autonomy led to difficulties in the recruitment due to the four-month residential training programme. Women’s ability to influence and make decisions within their households, therefore, needs to be considered from a human resource perspective.

It is vital to challenge the patriarchal notion that men should make decisions about whether their female household members can work outside the home. Challenging this notion needs to occur in communities with community leaders and APE supervisors who may inadvertently reinforce this notion as they are often called upon to liaise with husbands in instances where spousal consent to work outside the home was sought. The values and ideals of the supervisors are likely to influence this discussion—necessitating a training module for supervisors in how to approach discussions that have important implications for women’s intra-household bargaining power. Work within communities with the help of community leaders may also help to gain husbands’ acceptance, but importantly, also to transform harmful attitudes that see men threatened by women’s earning power or employment status. Challenging patriarchal norms around livelihood decision-making should occur simultaneously alongside supporting and being responsive to the strategic interests of APEs in these settings [46].

The success of gender transformation within the community comes from an Indian CHW programme which conceptualised CHWs as activists to empower the poor and marginalised. This vision was carried through to selection, training, ongoing support and systems of accountability and remuneration and resulted in the empowerment of women in the community [48]. They could access information about their rights, partook in community decision-making and understood violence against women as a social rather than personal issue [48].

### Training on selection for community leaders

Nepotism in selection, which has also been shown in the Democratic Republic of the Congo [44], was linked to giving income-generating opportunities to regard to young men. This has implications on the sustainability of the programme as it was reported they often leave for better-paid opportunities. Community leaders lead the selection process. Therefore, it would be beneficial to conduct thorough training with them around the roles and responsibilities of APE, aims of the programme and the importance of clear accountability mechanisms at the community level. This will help to ensure all community members are consulted on the selection of candidates, open up more opportunities for female candidates and avoid investment in candidates who are not committed to the role.

### Remuneration and social security

Given APEs’ essential role in the health care sector, there is a duty to ensure that they are appropriately and adequately supported by the health sector once through the recruitment process. Studies in Zimbabwe, Uganda and Mozambique have linked low and irregular pay and increasing workloads to poor retention and motivation amongst CHWs [15, 44]. Our results demonstrate a clear sentiment of APEs who felt conflicted—happy in their roles but also struggling to support their families within the realities of the working environment that sees increasing tasks and delayed subsidies. In particular, female APEs spoke movingly of feeling undervalued and unappreciated. Although there is no distinction in policy between male and females with regard remuneration, the experiences of women may have been influenced by their gendered positions which afford them relatively limited options for alternative sources of employment when compared with men, who were reported to travel to South Africa for mining opportunities.

Our findings corroborate literature from Mozambique and Ethiopia by Maes and Kalofonos [34] who used a life histories approach with CHWs to explore recruitment and retention. They report that CHWs feel undervalued and underpaid by the government—expressing frustration when organisations benefit from CHW work whilst the CHW’s aspirations for socio-economic advancement go unfulfilled. This sentiment was powerfully voiced by our female APEs describing themselves as ‘slaves of the ministry’. The study also critically notes that CHWs’ desire to earn money is beyond their own self-interest but in order to support, or in some cases, start a family [34]. This was a notion that was continuously expressed by both male and female APEs. Yet, despite their frustrations, APEs remain committed to their communities via a sense of duty to their community. Our respondents had internalised a common discourse of their work being priceless—and therefore unpaid—only remunerated with mental satisfaction. They suggest altruistic motives for remaining in the programme and used phrases like ‘serving our country’ and ‘there is no way a life is bought’. Similar sentiments have been expressed by volunteer CHWs in Nicaragua [43] and Ethiopia, where volunteers experienced psychological distress at not supporting their families, yet continued to volunteer out of obligation [49], highlighting the moral duty of care CHWs hold.

This highlights the complex issue of voluntary work. Voluntary work has been touted as a way to ensure intrinsic motivation [50] and sustainability; NGOs may also see their work as ‘ticking the sustainability box’ if it relies on the work of volunteers [41]. The reality of limited remuneration, however, is that can be a hindrance to empowerment for many CHWs across contexts [42] and may prevent CHWs from being taken seriously [41]. Many CHWs also risk impoverishment as they care for their communities at their own expense [49, 51]. Unpaid work is often taken on by those who can least afford it as it is viewed as a conduit into a paid position [12, 34, 41, 43], as voiced by our respondents who saw APE work as an entry point to a career in healthcare. Volunteerism is also inherently gendered, and women are often vertically segregated to unpaid, informal positions as they have more limited economic opportunities [52]. Recent findings from Afghanistan illustrate this, where male CHWs were praised for their volunteer work, but it was seen to be expected of women [9]. The assumption poor women can set aside time for voluntary work is problematic [53]. Further, labelling the work as voluntary reinforces the perception that women have domestic duties they need to work around [1] and bolsters the ‘double burden’ women face of juggling paid work with family responsibilities as expressed by our respondents.

In the Mozambican context, volunteerism may reinforce harmful gender norms as men quit for higher-paid positions, but women remain in the role suggesting their labour is cheaper and less valuable. However, care needs to be taken if moving to formalised employment. In India, a reported benefit of keeping the CHW role voluntary is that educational requirements could be relaxed for selection [10]. This would need to be thought through in the Mozambican context of interrupted education as a result of war and women’s lower level of literacy. Further, alongside offering formal employment needs to be strict selection criteria to ensure it is not just men who get selected for paid work, reinforcing gender power relations.

Our findings also linked limited autonomy over livelihood decisions to the inadequate financial gain from the role. Some husbands deemed the role of little value due to the minimal contribution to the household economy. Sen [54] describes how women’s contribution to the household may be diminished by gendered ideologies that characterise women’s income as ‘supplementary’ to that of the male ‘breadwinner’. Intra-household bargaining positions have also been shown to be influenced by an individual’s ability to pay, or perceived contribution to household livelihoods, amongst community members in Ghana [46]. Outside earnings therefore provide women with psychological and practical leverage and increase their decision-making power by increasing their perceived contribution to households [54].

The call for greater subsidies and formal employment rights, including social security, from the APEs should be heeded. Working at the lower end of the health system hierarchy, APEs are vulnerable to poverty and are overcoming damaging past events involving famine and conflict [34]. The provision of fair wages reduces food insecurity and helps progress towards Sustainable Development Goal eight for decent work and economic growth. It can provide men, but importantly women, an entry point into the formal work sector and employment rights such as insurance. Further, payment helps APEs to secure their livelihoods, contributing to empowerment - which has been argued to be an essential prelude to CHWs being committed to, and effective in, enacting their roles as health promoters and agents of change [42]. Formally paying both male and female APEs may also pay dividends to the broader health agenda and contribute to the economic development of the country- with; maternal income has been shown to lead to improved child health, one of the aims of the APE programme [45, 55].

### Further training opportunities, scholarships and career progression

Some younger female APEs spoke of their choices in choosing domestic work over APE work, but others felt limited in their choices due to their educational level. They articulated their desire to further their education in order to make them eligible for formal employment, better their futures and receive the recognition from the health system they felt they were owed. Developing further training opportunities, building a career structure and allocating sponsored places in higher education schemes for those eligible may be one way to further the transformation of this cadre and contribute to the economy and health workforce. A recent review of government reports by Percival et al. found no evidence that the Ministry of Health prioritised gender equity in its overall human resource strategy and that the promotion of women was not part of the recruitment process for the APE programme [25]. This highlights a key gap to address within the APE policy; however, the capacity of the health sector to absorb human resources trained in health will need to be considered and alternative pathways under ‘community health’ included. Success in increasing the number of women in supervisory roles was shown via gender-transformative programming by the President’s Malaria Initiative Africa Indoor Residual Spraying Project across African settings. Giving hiring priority to qualified women applying for supervisory positions helped to increase the percentage of women in supervisory roles from 17% in 2012 to 46% in 2015 [56]. Providing further career opportunities for eligible APEs may also encourage the aspirational younger generation to join, impacting the sustainability of the programme and eventually increasing the number of qualified health workers in the country. This may also particularly benefit women to progress their education and help to transform broader societal norms.

### Provision of health posts

Our research revealed interesting findings around policies on the place of work that have gendered implications for women. Policy dictates that the APEs do not operate out of a health post, but within communities—often using their own or community members’ homes. These policies however disproportionately impact on female APEs who are often not the primary decision-makers within their homes—if they treat clients out of their homes, they may be subject to family disapproval. This disapproval can lead to attrition, but it may also place women at risk of domestic violence, as voiced by a male APE. Violence can be considered one of the ‘most graphic expressions of unequal household power relations’ [57] and can further limit women’s autonomy. Provision of a dedicated health post to work out of is essential to improving the safety of female APEs and legitimacy and recognition of the cadre.

### Study limitations

Our study has some limitations that need to be considered; firstly, whilst we set out to explore the reasons behind the low levels of female participation in the APE programme, in Maputo Province, the ratio of women was reported to be much higher than is seen in the North and Central zones of the country. As this is a qualitative work, our findings cannot be generalised to the rest of the country but provide important insights into some of the considerations in recruitment and retention of male and female APEs. Secondly, it would have been beneficial to hear not just from current APEs but from those who had left the programme to explore their lived experiences and the reasons behind their attrition. The sample size of the single key informant was small but adds depth of knowledge and insight into the national context. Another potential limitation was the need to combine the male and female APEs into one FGD due to the limited numbers of APEs in Moamba. We feared this may have created some gendered power dynamics. However, the APEs presented themselves as a team and spoke openly about personal and community gender issues, and we elicited some rich responses this way. Finally, it is important to consider our position as researchers and the impact that our position as outsiders of the community, living in Maputo city and beyond, may have had on respondents. Despite this, participants generally seemed open and willing to talk to us and wanted to share their stories to enact change.

## Conclusion

Gender, age, geographical location and marital status all converge to influence the recruitment and retention of APEs within the Mozambican context. Research on health systems often focuses on social determinants, such as gender, as isolated factors. Nevertheless, our results highlight there is a need to investigate and understand how different axes of power intersect to create multiple identities. Data on APEs disaggregated by various axes of inequity will be crucial to conduct intersectional analyses in order to support informed decisions in APE policy-making at the national level. Supporting the recruitment of women into the role in line with government targets needs to be accompanied by supportive policies to ensure individuals are not recruited as cheap labour and their unique needs are met. Further, the extent to which APEs can bring their embedded knowledge into policy-making demands attention. This should be accompanied by consideration of existing power structures to ensure APEs have the agency to be heard and hold governing bodies accountable. Policy change must be underpinned by efforts to ensure women are not restricted by the current patriarchal norms within communities, as well as fair remuneration for the cadre. This will contribute to the empowerment of this cadre and redress women’s subordinate position in household decision-making—ensuring they have equal opportunities to enter the APE programme if they so wish.

## Data Availability

The datasets generated and/or analysed during the current study are not publicly available due to the anonymity of the participants but are available from the corresponding author on reasonable request.

## Appendix 1

### IDI guide with APEs

Introduction: Thank you for taking the time to speak to me. I would like to understand a little bit more about the recruitment of male and female APEs, reasons for leaving and how the health system can better support APEs of both genders. If you do not want to answer any of the questions in the interview, please let me know and we can move on to something different. If any of the questions are unclear, please tell me and I am happy to ask them in a different way.

**Introductory questions/background**

- Age
- Education/years of school
- Can you describe your household?
  - Who do you live with?
  - What are your duties around the house/What responsibilities do you have at home?
    - Does anyone help with these duties?

**Reasons for becoming an APE**

- Have you always lived in this community?
  - If no, where did you live before?
    - Why did you move?
    - How was the community different to this one?
- How long have you been an APE?
- Why did you become an APE?
- What do you think are the main reasons that people become APEs?
  - Do you think these reasons are the same for both men and women?
- What are the main things you like/dislike about your role? Why?
- Have you ever wanted to leave or do anything else?
  - If yes, what is currently stopping you from engaging with these activities?
- How well do you feel supported by the health system?
  - How could they support you better?
  - Would you say the same for both male and female APEs?
  - What do you think they are doing well at?
- Do you feel you are offered the same support as your male/female counterparts?
  - Are there more definite roles between genders?
  - Is the pay grade the same?
  - Probe: Are the reasons different between genders?

**Training**

- Can you tell me a little about the training that is offered to APEs?
- I understand the training is carried out in a different village—did this have any bearing on your decision to become an APE?
  - Was your spouse supportive of this?
  - Do you think this ever causes problems for male/female APEs?
    - If so, how does it differ between the sexes?
  - What if the training was run in shorter intervals or closer to home, how might this change things?
- Are you offered continuous training?
  - When was the last?
  - What was the subject of the training?
  - Was it relevant to what you do?
  - Are there any gender issues relating to the training?
    - If so, how?
- Do you think the training offered in the community would be helpful?
  - If not, do you think separate training based on gender would be beneficial at all?
- Are there any changes you would make with regard to the training which you feel would improve your role/efficiency—help you do your job better?

**Supervision**

- How is your supervision conducted?
- Can you describe your relationship with your supervisor?
  - How does your relationship impact your work?
- Is your supervisor male or female?
  - If male, have you ever had a female supervisor?
  - Do you think their gender has any effect on your relationship?
  - How may things be different if you had a supervisor of the opposite gender?
- Outside of routine reporting, how do you report to the health system?
  - Do you think this is generally similar to both male/female colleagues?

**Policies/guidelines**

- What about guidelines, do you have clear guidelines around the training and supervision?
  - Do they support you as a woman/man specifically?
    - How specifically?
    - How could they improve?
    - How do you think they differ for your colleagues of the opposite sex?
    - Do you think there is any need for differing guidelines for male and female APEs?
  - Is there anything you would like to see in the guidelines to help support you in your day-to-day?

**Attrition**

- I would like to ask about why people leave their roles—from your experience or in your opinion, what do you think are the main reasons people stop working as an APE?
- What could the health system do to change this?
  - Or do you think there are more personal reasons people leave—i.e. family commitments?
- Do you think there are different reasons for males leaving than for females?
  - If required, can you expand on this?

Thank you so much for the time. Is there anything else you want to share with me?

## Appendix 2

### FGD guide with APE

Introduction: Thank you for taking the time to speak to me. I would like to understand a little bit more about the recruitment of male and female APEs, reasons for leaving and how the health system can better support male/female APEs. If any of the questions are unclear, please tell me and I am happy to ask them in a different way. You are free to leave at any time.

**Reasons for becoming an APE**

- What do you think are the main reasons that people become APEs?
  - Do you think these reasons are the same for both men and women?
- What are the main things you like/dislike about your role?
- Have you ever wanted to leave or do anything else?
  - If yes, what is currently stopping you from engaging with these activities?
  - Probe here.
- How well do you feel supported by the health system?
  - What are they doing well/How could they support you better?
  - Would you say the same for both male and female APEs?
- Do you feel you are offered the same support as your male/female counterparts?

**Training**

Can you tell me a little about the training that is offered to APEs?

- I understand the training is not in the community—did this have any bearing on your decision to become an APE?
  - Do you think this ever causes problems for male/female APEs?
  - Why?
- Do you think the training offered in the community would be helpful?
  - What about separate training by gender?
- Are there any changes you would make with regard to the training which you feel would help you do your job better?

**Supervision**

- Can you describe your relationship with your supervisor?
- Is your supervisor male or female?
  - Do you think their gender has any effect on your relationship?
  - How may things be different if you had a supervisor of the opposite gender?

**Attrition**

- I would like to ask about why people leave their roles—from your experience or in your opinion, what do you think are the main reasons people stop working as an APE?
  - Probe here.
- Do you think there is anything the health system could do to change this?
- Do you think there are different reasons for males leaving than for females?

Thank you so much for the time. Is there anything else you want to share with me?

## Appendix 3

### IDI guide with supervisors

Introduction: Thank you for taking the time to speak to me. I would like to understand a little bit more about the recruitment of male and female APEs, reasons for leaving and how the health system can better support male/female APEs. If any of the questions are unclear, please tell me and I am happy to ask them in a different way. You are free to leave at any time.

**Reasons for becoming a supervisor**

- What are the main things you like/dislike about your role as a supervisor?
- Can you describe how APEs in your community were selected?
  - What do you think are reasons people want to do this role?
  - Do you think these reasons are the same for men and women?
  - Do you think men and women are given equal opportunity to be selected as an APE?
- Once in the role, do you feel the health system offers the same support to male and female APEs?
- What else could the health system do to support APEs?
  - Would you say the same for both male and female APEs?

**Training**

Can you tell me a little about the training that is offered to APEs?

- I understand the initial training is outside the community—do you think this has any bearing on people’s decision to become an APE?
  - Do you think this ever causes problems for male or female APEs specifically?
  - How do you think the health system might address this?
- Are there any changes you would make with regard to the training that you feel would improve APEs’ efficiency/performance?

**Supervision**

- Can you describe your relationship with the APEs you supervise?
- Do you have a different relationship with your male and female APEs?
  - Do you find any differences in the performance between the sexes?
  - Are there any cultural issues or ethnic barriers related to gender?
- If male: Are there many female supervisors in your team?
  - If no, why do you think that is?
  - If yes, do you think they have a different relationship with male/female APEs? Why?

**Policies/guidelines**

- Are you aware of any guidelines on supervision?
  - Are they gender-specific?
    - If so, please expand.
  - Is there anything you would like to see in the guidelines to help support you in your day-to-day as a supervisor?

**Attrition**

- From your experience, what do you think are the main reasons APEs stop working?
- Do you think there is anything the health system could do to change this?
- Do you think there are different reasons for males leaving than for females?
  - Explain.

Thank you so much for the time. Is there anything else you want to share with me?

## Appendix 4

### IDI with community leader guide

Introduction: Thank you for taking the time to speak to me. I would like to understand a little bit more about the recruitment of male and female APEs, reasons for leaving and how the health system can better support APEs of both genders. If any of the questions are unclear, please tell me and I am happy to ask them in a different way. You are free to leave at any time.

**Reasons for becoming an APE**

- What do you think are the main reasons that people become APEs?
  - Do you think these reasons are the same for both men and women?
  - Can you describe how the APE was selected in this community?
    - Why were they selected?
    - Probe: Do you prefer a male or female and why?
    - If male: Were there female candidates?
    - Are women and men given equal opportunity for selection?
- Do you think the health system supports APEs?
  - How do you think they could support them better?
- Do you feel the same level of support is offered to male and female APEs?
- Is there any difference in the performance of APE by sex?

**Training**

- I understand the initial training on becoming an APE is carried out in a different village—do you think this has any bearing on people’s decision to become an APE?
  - Do you think this ever causes problems for male or female APEs specifically?
    - If so, how does it differ between the sexes?
  - Do you think there is enough of a career opportunity for APE?

**Attrition**

- I would like to ask about why people leave their roles—from your experience, what do you think are the main reasons APEs stop working?
- Do you think there is anything the health system could do to change this?
- Do you think there are different reasons for males leaving than for females?
  - Please explain.

Thank you so much for the time. Is there anything else you want to share with me?

## Appendix 5

### KII Mozambique

Introduction: Thank you for taking the time to speak to me. I would like to understand a little bit more about community health workers (CHWs) and if/how gender roles and relations affect their work and experiences and if so the extent to which this is considered in policy and practice. If you do not want to answer any of the questions in the interview, please let me know and we can move on to something different. If any of the questions are unclear, please tell me and I am happy to ask them in a different way.

**Intro questions:**

- Occupation:
- Organisation:
- Can you tell me a bit about your experience with CHW programmes or policy development?

**Supervision:**

- Can you describe the APE programme?
  - Who works best at which roles (by gender)?
  - Why do you think the pattern is like that?
- In Mozambique, what is the ratio of male to female CHWs?
  - Why is it like that?
  - Do you know why there is a current target to recruit more female APEs?
    - Who was involved in setting that target?
    - What are the reasons/driving force for that target? What is it about gender that is important to the APE programme?
  - Why do you think communities choose male/female CHWs?
    - Probe: Intra-household dynamics that drive that.
- How does this compare to the ratio of male to female supervisors?
  - What do you think are the main reasons for this?
  - What impact do you think this has?
  - Do you see this changing through policy?
- Do you think gender issues impact on the delivery of services in your context?
  - Probe: In what ways?
  - Do you think supervisors are aware of the importance of gender issues as part of the delivery of services?
- Do you think gender issues impact on CHWs’ relationship with the health system?
  - Probe: In what ways?
  - Do you think supervisors are aware of the importance of gender for the relationship with the health system?

**(New) policy development**

- Can you describe the process of new CHW policy development?
  - Who are the key players involved?
  - Was it intersectoral?
  - Any representation from the gender department/women’s groups/movements?
    - If so, are there mechanisms for carrying out intersectoral efforts aimed at improving health?
  - To what extent do CHWs or supervisors bring their knowledge into policy development?
- When policy is being developed, what information is available?
  For example, is information available on the sex breakdown of CHWs, including the areas in which men and women work, remuneration levels, representation at decision-making levels and length of employment, at both the national and local levels.
- Can you tell me about the revitalised program for APEs?
  - In what way do you think conflict impacted the revitalised policy?
  - How did gender play out in this?
  - Do you think gender was considered whilst the revitalisation happened?
  - If not, why not? Do you think it was a lost opportunity to consider gender?
  - If yes, in which ways—probe here.
- Was/Is there any impetus to include gender in policy development?
  - Where is the impetus to include gender coming from? Is anyone bringing it to policy development table?
    - If not, why do you think that is the case?
    - If so, were they always listened to?
    - What has changed in the last 5/10 years?
- What is your feeling on women’s influence on local agendas?
  - Has this changed over time?
  - If so, why do you think that is the case?
  - If not, how do you think this could change?
    - What would be needed to bring about this change?
- What do you think is needed to develop a new gender-specific or transformative policy?
  - Probe: Is it an attitude change that is needed or more of a structural change?
- From your experience, what is the key thing you could suggest to ensure CHW policy can be more gender-specific?

**Current guidelines/tools**

- How would you describe the current status of gender in CHW policy is in your country?
  - Is gender explicitly considered in CHW policy?
    - If so, in what way? Does the policy recognise the need to identify and address the differences between women and men with regard to needs, knowledge, opportunities and compensation?
    - If so, is this policy followed and is it effective?
    - Are you aware of any of the main indicators used relating to this policy?
- From your position, what is the general feeling around CHWs and gender-specific policy?
  - Is there much debate or discussion around it?
    - If yes, what is being said?
    - If no, why do you think that is, why is no one championing it? Is there any intersectoral collaboration with the gender departments/ministries, etc.
- In your context, do you think CHWs and health systems staff (e.g. supervisors) routinely receive training to develop awareness of the differing situations and needs of women and men?
  - If not, is there any debate about gender?
  - If yes, can you give any examples?
  - If yes, is this training competency taken into account in supervision and performance evaluation?
- Are you aware of any policies that support equal opportunity for women and men in recruitment, training and promotion in employment?
  - If so, is this policy followed and is it effective?
  - Are you aware of any of the main indicators used relating to this policy?
- Are there equal opportunities for male and female CHWs to progress their careers?
  - Has an explicit policy been formulated that supports equal opportunity in employment and specifically mentions gender equality?
  - If so, how did this come about? Are there indicators/targets?
  - If not, why do you think this is the case?
  - How do you think this could change?

**Implementation**

- Are there any action plans in place to implement existing policy around CHW and gender?
  - For example, something that would include a situation assessment, objectives, and indicators of gender equity?
  - If not, then ones to identify gender differences?
  - If so, how much progress is made?
  - Main challenges?
- If there is no policy, how likely do you think it is that a policy like this would be introduced, or how likely do you think it could be implemented?
- Is there a HIS that could support HR management?
  - If not, do you think this would be feasible in your context?
  - If yes, how is it used generally
    - Is it used to monitor compliance with the gender equality policy
    - Can it provide sex-disaggregated information?
    - Is it ever used to support timely decision-making?
- What would be the one thing you think would need to change/or what needs to keep occurring to ensure effective implementation of gender-sensitive policies?

Thank you so much for the time. Is there anything else you want to share with me?

## Abbreviations

APE: Agentes Polivalentes Elementares
CHW: Community health worker
FDG: Focus group discussion
IDI: In-depth interview
NGO: Non-governmental organisation
